# Evaluation of schistosomula crude antigen (SCA) as a diagnostic tool for *Schistosoma mansoni* in low endemic human population

**DOI:** 10.1038/s41598-021-89929-3

**Published:** 2021-05-18

**Authors:** Oyetunde Timothy Oyeyemi, Camila Amormino Corsini, Gustavo Gonçalves, William de Castro Borges, Rafaella Fortini Queiroz Grenfell

**Affiliations:** 1 Department of Biological Sciences, University of Medical Sciences, Ondo, Ondo State Nigeria; 2 The Laboratory of Diagnosis and Therapy of Infectious Diseases and Cancer, René Rachou Institute, Oswaldo Cruz Foundation (Fiocruz), Belo Horizonte, Minas Gerais Brazil; 3 The Laboratory of Enzymology and Proteomics, Federal University, Ouro Prêto, Minas Gerais Brazil

**Keywords:** Immunology, Microbiology, Zoology, Biomarkers

## Abstract

The study aimed to determine the potential of schistosomula crude antigen (SCA) as a diagnostic target for anti-*S. mansoni* antibody detection. Cercariae were transformed into schistosomula, homogenized through sonication, and then centrifuged to obtain the SCA. SCA was evaluated using ELISA and dot blots immunoassays on 30 *S. mansoni* infected sera samples obtained from chronic patients and 30 non-infected humans’ sera samples. Either Kato-Katz or saline gradient method or both were employed as the diagnostic reference. Dot blots immunoassay was further performed on protein eluted from 10 to 12 kDa immunoreactive band identified by Western blot analysis. The area under the ROC curve was 0.95 (AUC 0.95, CI 0.88–1.01, *p* < 0.0001). The sensitivity and specificity of SCA-ELISA and dot blots assays were 96.67% and 86.67% respectively. The human IgG-specific response against SCA was significantly higher in *S. mansoni* infected individuals (OD = 0.678 ± 0.249) compared to the non-infected population (OD = 0.235 ± 0.136) (*p* < 0.0001). Our study showed that SCA and its 10–12 kDa component could be useful as diagnostic tools for chronic schistosomiasis.

## Introduction

Schistosomiasis is a disease that raises a significant public health concern due to the burdens associated with it. It is a waterborne disease caused by *Schistosoma* spp. The parasite utilizes specific freshwater snail for the development of its infective form. A global estimate in 2018 showed that about 230 million people required schistosomiasis preventive treatment^1^. *Schistosoma mansoni* is the species causing human schistosomiasis in Brazil. Schistosomiasis is endemic in 19 of the 27 states in Brazil with over 70% of cases from the Northeast region^2^. An estimated 1.5 million individuals were infected with *S. mansoni* and 42.9 million people were at high risk of infection^3^. The high prevalence of schistosomiasis and associated severe morbidities are largely due to persistent exposure to the source of infection which is usually contaminated water bodies especially in areas with inadequate water supply^4^.

Traditionally, *Schistosoma* infection is determined by microscopic detection of parasite eggs in human feces or urine. In *S. mansoni,* the sensitivity of microscopy is low with decreased number of eggs^5,6^. To overcome the diagnostic deficiency of microscopy associated with low intensity of infection, a commercially available indirect haemagglutination test (IHA) using erythrocytes coated with *S. mansoni* adult worm antigens and in-house ELISAs to detect antibody against *S. mansoni* egg antigens (SEA) in patient’s blood was widely used^7^. The application of this tool is, however, limited to non-endemic regions of developed countries.

Other serological diagnostic methods have been extensively explored for the diagnosis of schistosomiasis in the developing world. These have been widely advocated in areas or populations with low transmissions of infection^8–11^. Studies have explored the anti-*Schistosoma* antibodies produced in response to the antigens derived from the different stages of the parasite. The notable stages often used in serological assays are; the eggs, cercariae, schistosomula, adult worms, and proteins associated with these stages^6,11–13^.

Currently, the available antibody detection immunoassays make use of antigens derived from the eggs and adult worms^14,15^. The application of schistosomula antigens as an immunodiagnostic method for early detection of *Schistosoma* infection has been previously explored among travelers and acute patients^10,12^, however, little is known about its suitability for evaluation of chronic infection.

Our study aimed to explore the immunodiagnostic potential of *S. mansoni* schistosomula crude antigen (SCA) among chronic patients with low intensity of infection in endemic communities in Brazil. To achieve this, the diagnostic potential of the antigen was ascertained by detection of anti-*Schistosoma* IgG by enzyme-linked immunosorbent assay (ELISA) in patients’ sera. We also determined the antigenicity of schistosomula antigens by Western blot. Besides, we validated the immunoreactivity through dot blots immunoassay using SCA and protein eluted from the immunoreactive protein band from sodium dodecyl sulfate–polyacrylamide gel electrophoresis (SDS-PAGE) identified by Western blot.

## Results

**Figure 1:**
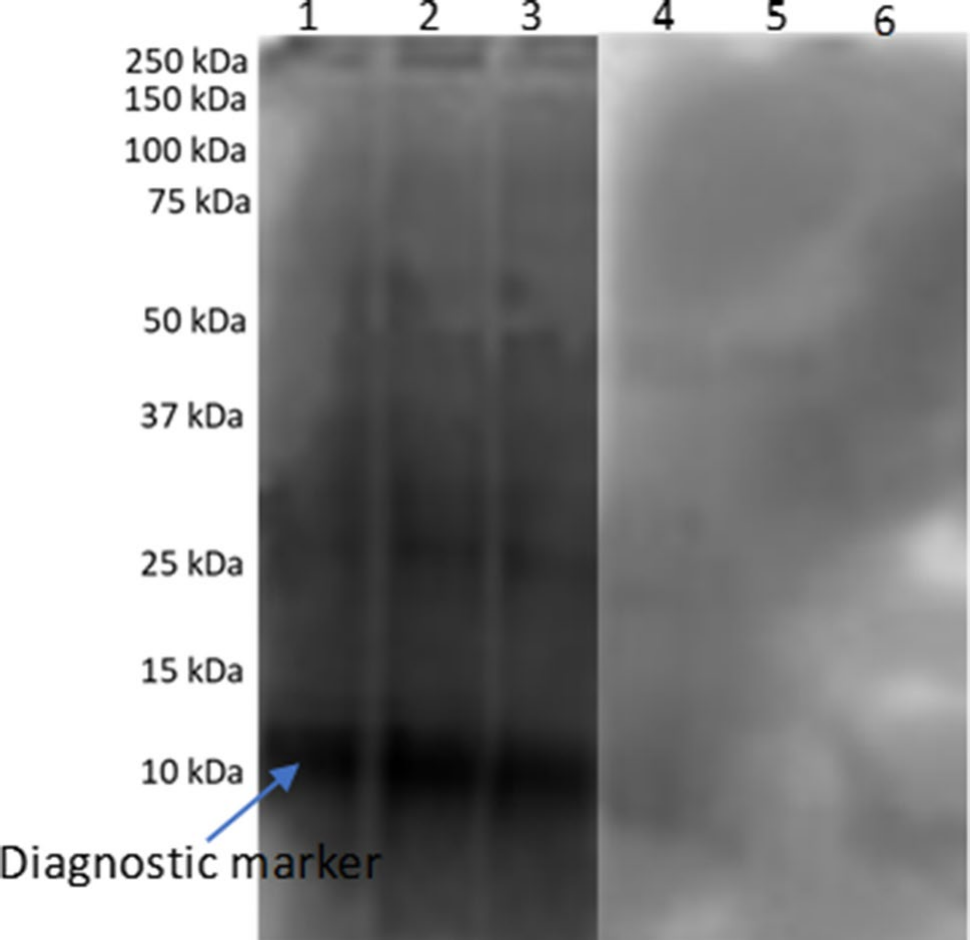
Western blot analysis of SCA and *S. mansoni* positive and negative sera.

**Figure 2:**
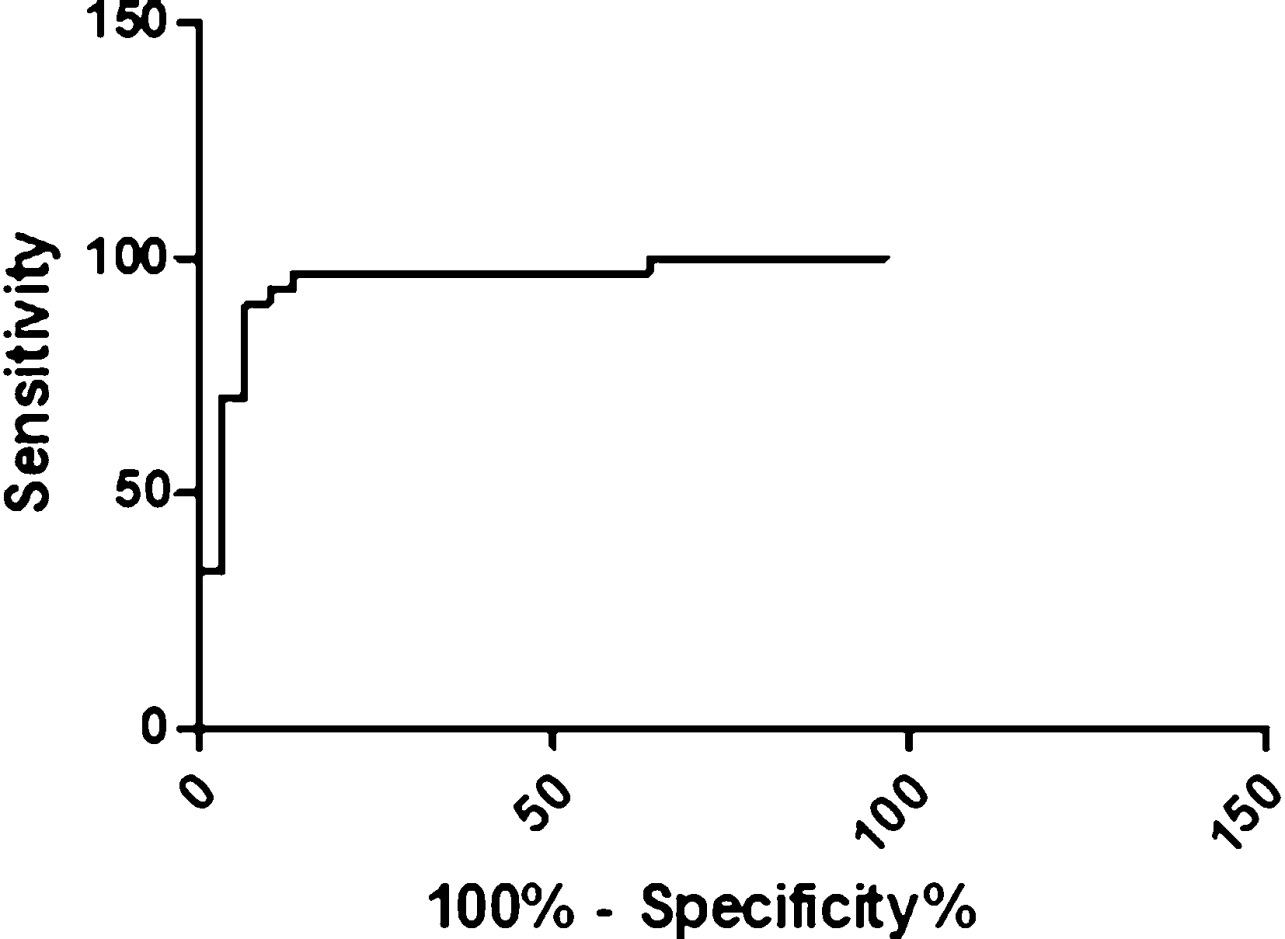
Receiver operating characteristic curve of ELISA immunoassay of SCA.

The immunoreactive protein bands were recognized by the pooled infected sera from *S. mansoni*. Importantly, protein band size 10–12 kDa showed an *S. mansoni* specific prominent immunoreaction in Western blot (Fig. 1; see also the supplementary file). The protein contents of SCA and excised immunoreactive protein bands were 4.051 and 9.781 μg/μl respectively. The cut-off value determined for ELISA assay using 30 *S. mansoni* positive and 30 negative sera samples was 0.288. The area under the ROC curve was 0.95 (AUC 0.95, CI 0.88–1.01, *p* < 0.0001) (Fig. 2).

**Figure 3:**
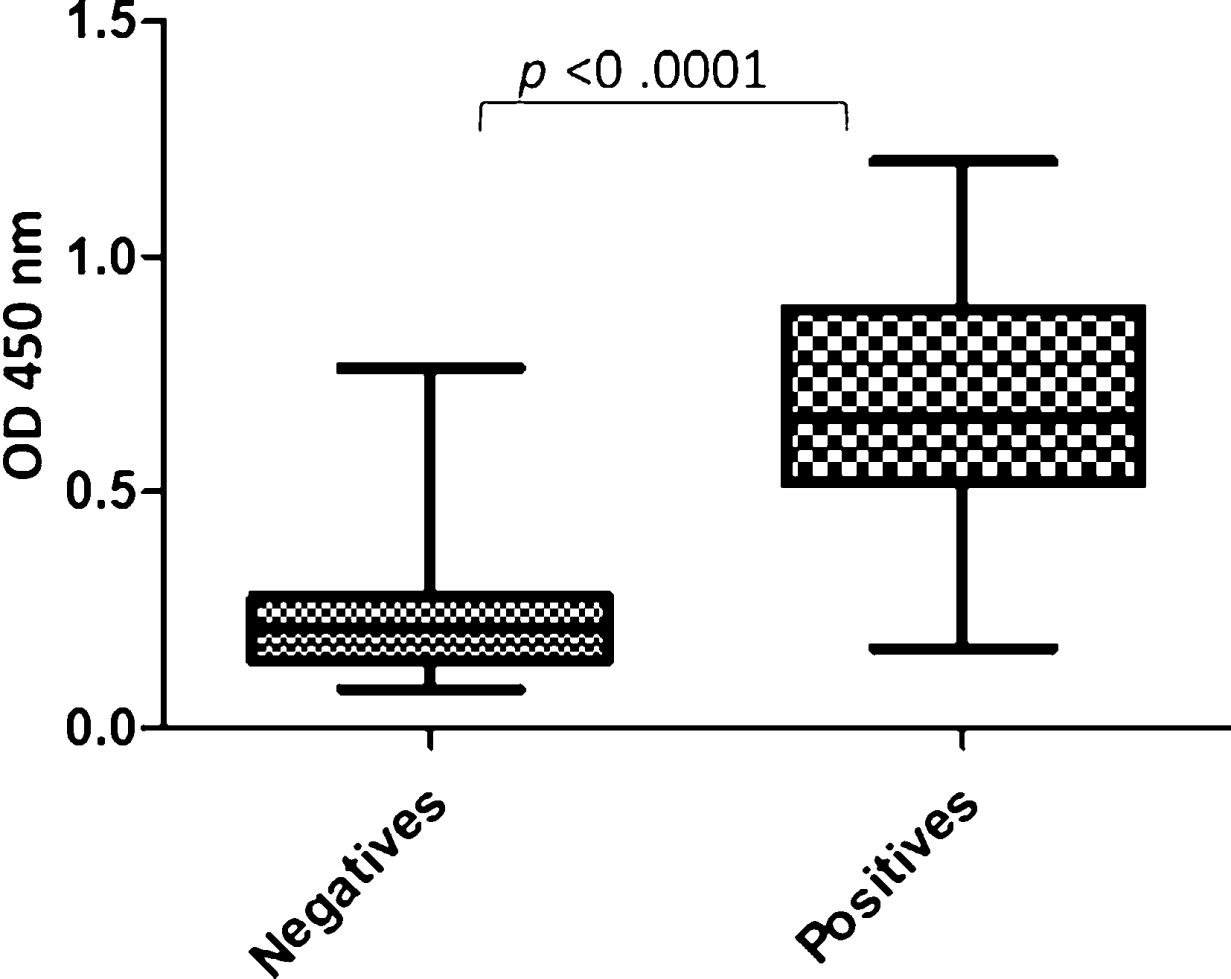
Antibody production in relation to *S. mansoni* infection status.

The sensitivity and specificity of SCA in diagnosing *S. mansoni* infected individuals using ELISA assay were 96.67% (CI 82.78–99.92%) and 86.67% (69.28–96.28%) respectively (Table 1). The overall diagnostic accuracy of SCA 91.67% (81.61–97.24%) was higher than that of SEA 88.33% (77.43–95.18%) but slightly lower than that of SWAP 93.33% (83.80–98.15%). The human IgG-specific response against SCA was significantly higher in *S. mansoni* infected individuals (OD = 0.678 ± 0.249) compared to the non-infected population (OD = 0.235 ± 0.136) (*p* < 0.0001) (Fig. 3).

**Table 1:** Comparison of *S. mansoni* diagnostic potential of SCA with ELISA diagnostic references.

Antigens	Accuracy % (CI 95%)	Sensitivity % (CI 95%)	Specificity % (CI 95%)	AUC
SCA	91.67 (81.61–97.24)	96.67 (82.78–99.92)	86.67 (69.28–96.28)	0.95 (0.88–1.01)
SEA^a^	88.33 (77.43–95.18)	90.00 (73.47–97.89)	86.67 (69.28–96.24)	0.90 (0.82–0.98)
SWAP^a^	93.33 (83.80–98.15)	90.00 (73.47–97.89)	96.67 (82.78–99.92)	0.96 (0.91–1.00)

SCA; schistosomula crude antigen, SEA; soluble egg antigen, SWAP; soluble worm antigenic preparation, AUC; area under curve.

(^a^) is ELISA diagnostic references—same sera samples used for SCA, SEA and SWAP.

Of the 30 positive samples diagnosed, the number of samples that came out as true positive was 29 while 4 were observed false positive. In the 30 non-infected sera samples, 26 of the samples came out as true negative while 1 was observed as a false negative. The number of true and negative results in SCA-ELISA relative to SEA and SWAP was presented in Table 2. SCA-ELISA showed Kappa index 0.83 when compared to 20 KK slides or SG or both. The Kappa index of SCA-ELISA compared to SWAP-ELISA was 0.77.

**Table 2:** Number of true and false result in SCA-ELISA in comparison with SEA and SWAP.

Proteins	TP	FP	TN	FN
SCA	29	4	26	1
SEA	27	4	26	3
SWAP	27	1	29	3

SCA; schistosomula crude antigen, SEA; soluble egg antigen, SWAP; soluble worm antigenic preparation.

TP—true positive, FP—false positive, TN—true negative, FN – false negative (n = 30).

**Figure 4:**
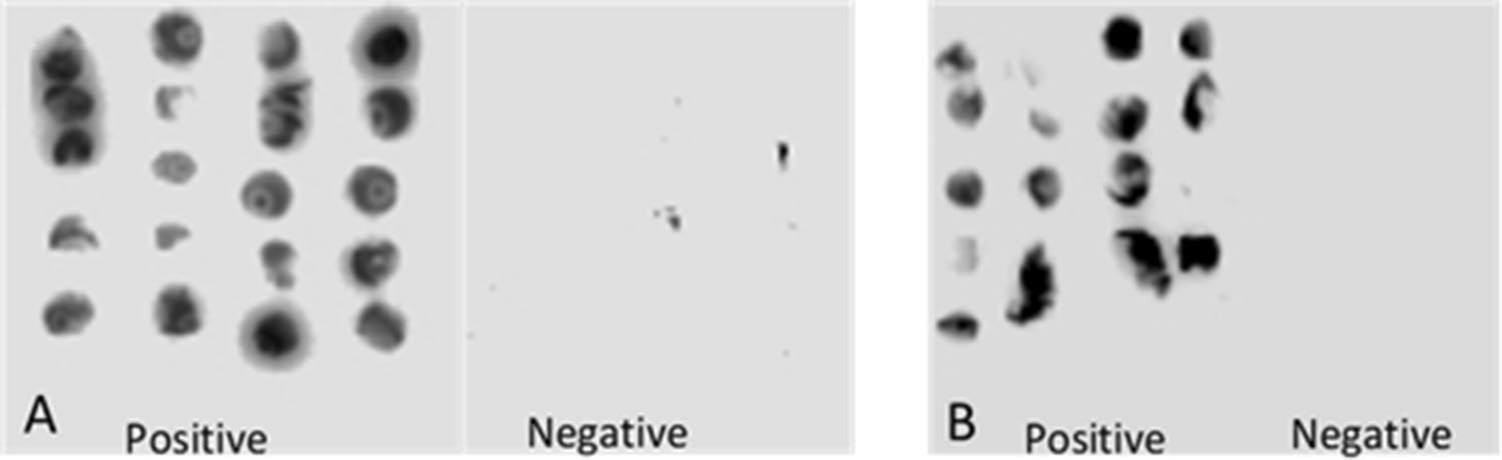
Dot blot assays of SCA (**A**) and immunoreactive protein band (10–12 kDa) (**B**).

Similar results with sensitivity 96.67% (CI 82.78–99.92%) and specificity 86.67% (69.28–96.28%) were recorded for dot blots analyses of SCA against *S. mansoni* positive and negative sera. Like in ELISA, 26 samples were observed as true negative while 1 was observed as a false negative. The dot blots images of SCA and the excised immunoreactive protein band (10–12 kDa) captured using chemiluminescence detection were presented in Fig. 4. The dot blot assays of SCA (Fig. 4A) and the immunoreactive protein band (10–12 kDa) (Fig. 4B) showed reaction with the positive sera but no reaction was observed in the negative-control sera (see also the supplementary file).

## Discussion

Schistosomiasis inflicts serious morbidity on the human host. Eggs deposition in blood vessels draining the intestine as in *S. mansoni*, during the chronic stage of the infection, is responsible for the morbidity of the disease. So, a good diagnostic test should be efficient to detect the infection as early as possible after the onset of infection^6^. An early diagnosis especially in acute infection as in the case of floating population or travelers would facilitate early treatment and interrupt the severe morbidity associated with the disease progression. During exposure to *Schistosoma* infection in human hosts, the immune systems respond to four main stages in the parasite’s life cycle: the infective stage cercariae, the young worm schistosomula, adult worms, and the eggs^13^. Since cercariae and schistosomula are the two early stages the host is exposed to, invariably, antigens expressed by these stages are potential diagnostic biomarkers for early infection. Besides the early diagnostic advantage of schistosomula antigen, it likely retains some of its protein in adult worms, thus, it can also be suitable for the diagnosis of chronic infection. It is therefore not surprising the excellent diagnostic results we recorded in the present study that made use of individuals with chronic infection.

The identified 10–12 kDa immunoreactive protein in our study was much smaller than 62 kDa protein that stimulated CD4^+^ Th cells and 37 kDa diagnostic protein known as the major egg antigen (MEA) from SEA^16,17^. Although other immunoreactive proteins of higher molecular weight (25–37 kDa) were identified in the Western blot assay, the intensity of the reaction was weaker and these proteins showed no consistency in the subsequent dot-blot immunoassay performed.

The area under a ROC curve determines the overall ability of the SCA to discriminate between *S. mansoni* infected and non-infected individuals. The AUC value observed for SCA compared favorably well with SWAP and was better than SEA which is often used as diagnostic reference standards for ELISA. The SCA-ELISA protocol was properly optimized as a marked difference was noted in the antibody titers between sera from *S. mansoni* infected and non-infected individuals. The 96.7% sensitivity in our study was better than 89.7% recorded from cercarial transformation fluid reported by El Aswad Bel et al.^18^. However, the latter study recorded a better specificity compared to our study. In comparison with another study on *S. mansoni* SEA^19^, our study showed a slightly improved sensitivity but significantly lower specificity. This discrepancy in diagnostic performance could be due to variation in the intensity of *Schistosoma* infection in the positive-control sera.

It is important to state that this study was designed to detect chronic infection since the communities the sera were sourced from are endemic and the subjects are permanent residents. Although the acute infection was not evaluated, SCA can proffer a diagnostic advantage over SWAP in such a situation. Also, in case of re-infection after the establishment of chronic disease (after drug treatment), SCA could be more suitable to detect a new infection than SWAP and SEA. This therefore could contribute to the slightly higher sensitivity recorded in SCA.

Practically, it appeared SCA effectively diagnosed sera of individuals with adult worms or those who harbor *Schistosoma* eggs just as SWAP and SEA. Although no reference has possibly supported this, a previously unreported study by our group showed some similarities in protein patterns in the gel-electrophoresis analysis of SCA and SWAP, thus, suggesting some common diagnostic proteins are present in the parasite’s different stages. So, proteins in SCA which can diagnose chronic disease in addition to its early diagnostic advantage can therefore be responsible for its impressive diagnostic performance. Similar to the methods adopted in this study, several studies have also explored the diagnostic potential of certain antigen preparation from the early stages of *Schistosoma* spp. against antibodies associated with the adult worms, or their eggs^19,20^. The high sensitivity of SCA could provide an alternative to the use of SEA and SWAP, which are difficult to prepare and generally not cost-effective.

The similar diagnostic performance of SCA dot blots when compared to ELISA is very desirable and this suggests its suitability for point of care (POC) cassette test. The same can be said of the impressive immunoreaction recorded with the diagnostic protein band component (10–12 kDa). An impressive point of care (POC) rapid diagnostic approach was previously achieved with *S. mansoni* cercarial transformed fluid in children age 5 to 12 years^21^.

One limitation of this study was the small sample size used for the SCA-ELISA immunodiagnosis. Also, the inability to elute an adequate amount of protein from the 10–12 kDA protein band limited the number of sera samples employed for the corresponding dot blot assay. We did not include in the evaluation serum samples from non-infected individuals living in endemic areas and sera from individuals infected with other helminthic infections. This may undermine the reliability of the results due to possible cross-reaction between SCA and other helminths.

Our study suggested that SCA and its 10–12 kDa protein component are potential diagnostic tools for chronic *S. mansoni* infection with low parasite intensity. However, a diagnostic protocol that will employ a gold standard that can differentiate acute from chronic infection may be more suitable for the evaluation of SCA as a target for anti-*S. mansoni* antibody detection. The preparation of SCA is easier and cheaper than that of SEA and SWAP. Therefore, where SEA or SWAP is not available, SCA can be adopted for immunodiagnosis of *S. mansoni*. Besides, to achieve a perfect diagnostic result, it can be used simultaneously with SWAP or SEA. This diagnostic approach may be suitable for acute and pre-patent infections which ordinarily pose a diagnostic challenge to SEA or SWAP. We recommend the consideration of the proposed components for descriptions when reporting development and/or evaluation of serological diagnostic methods^22^. Further studies to identify, purify, and recombinantly produce the immunoreactive protein are highly recommended. The application of the protein in the POC rapid diagnostic test approach is also recommended as it would aid in the quick identification of the infected population.

## Methods

### Source of human sera

Laboratory stored sera samples obtained from *S. mansoni* chronic individuals sourced from three rural communities; Pedra Preta, Tabuas, and Estreito de Miralta were used for the study. The samples were sourced between 2009 and 2014 and the areas fell within schistosomiasis low-endemic areas with a mean intensity of infection 12.2 ± 14.4 epg of fecal samples (range; 1–47 epg). The age range of the subjects was 1 – 79 years (33.8 ± 21.7 years). A combination of methods including; Kato-Katz (KK), saline gradient (fecal samples), point-of-care circulating cathodic antigen (POC-CCA using urine), and SWAP (sera) was used for the diagnosis of *S. mansoni.* Each subject provided a single sample that was used to make 24 KK slides (24 × 41.7 mg = 1 g) (Helm-Test, Biomanguinhos, FIOCRUZ, Brazil)^23^, and 2 procedures of saline gradient (SG) technique (2 × 500 mg = 1 g)^24^. The positive-control sera used for SCA-ELISA immunodiagnosis were selected based on positive KK and SG results, positive KK, positive SG, or if all the four methods are positive. Individuals with POC-CCA, SWAP or both without positive KK or SG results were excluded. So, the reference standard was a positive KK or SG or both. Sera samples from healthy individuals living in a schistosomiasis non-endemic area in Belo Horizonte, Minas Gerais, who had no history of previous *Schistosoma* infection, were used as the negative control (Age 21–70 years old)^16^. Fecal samples from these individuals were also screened for *S. mansoni* and other helminths by KK and SG. Sera which were positive to other helminths were excluded from the negative control. Those in the negative control group with positive ELISA-SWAP and POC-CCA results were excluded from the analysis. In all, 30 *S. mansoni* positive and 30 negative sera samples were used for all immunoassays.

### Preparation of the *S. mansoni* schistosomula crude antigen (SCA)

*Schistosoma mansoni* cercariae of LE strain were obtained from the Mollusc Rearing Facility, Lobato Paraense, René Rachou Institute, Fiocruz. Mineral water, beakers (500 ml), and conical tubes (50 ml) (for the mechanical transformation of cercariae) were chilled in an icebox before the start of the procedure. The pond water containing cercariae was carefully poured into a 500 ml chilled beaker and was further chilled on ice for another 1 h 30 min to reduce the parasite mobility. The pond water was carefully pipetted from the layer of cercariae that settled at the bottom of the beaker until about 50 ml of cercarial suspension remained. The cercariae were resuspended in the remaining water by gentle swirling and aliquots of the cercarial suspension were transferred into a 50 ml chilled conical tube. The beaker was rinsed with chilled sterile mineral water and the aliquot of the cercarial suspension was transferred into another 50 ml tube. The cercarial samples were centrifuged at 1200×*g* for 5 min. The supernatant was discarded and the cercariae pellets were resuspended in 10 ml of chilled RPMI-1640 culture medium. The parasites were washed two more times in the culture medium by centrifugation.

The parasite pellet was resuspended in 3 ml of chilled RPMI 1640 + Pen/Strep medium. The tubes were vortexed for 2 min to mechanically transform the cercariae by separating the bodies from the tails. The bodies and tails were transferred into a cell culture flask, topped up to 30 ml using the RPMI 1640 + Pen/Strep medium, and then incubated at 37 °C and 4% CO_2_ for 3 h. After incubation, the culture medium which now contained fully formed schistosomula and active tails were centrifuged. The supernatant was discarded and the parasite pellet was then resuspended in mineral water. Schistosomula settled at the bottom within 1–2 min while the motile cercariae tails were seen suspended within the water interface. The tails were removed and the process was repeated until all tails were removed. The suspension containing schistosomula was sonicated using six 10 s pulses on full power with 1 min on ice between each sonication^16^. The suspension was centrifuged at 20,000×*g* for 10 min. The supernatant was collected and was stored at − 20 °C until required. The protein concentration of the schistosomula crude extract was determined by Bicinchoninic acid assay (BCA) (ThermoScientific, Rockford, lL 61105 USA) and the quality of the extract was verified by 12% SDS-PAGE.

### Indirect enzyme-linked immunosorbent assay (ELISA)

Anti-schistosomula IgG antibody was determined according to the method described by Grenfell et al.^12^ with some modifications. Briefly, each well of the microtiter plate MaxiSorp Surface (NUNC Brand Products, Roskilde, DK) was coated with 100 µl of parasite extract diluted at 1 µg/ml in the carbonate-bicarbonate buffer and then incubated overnight at 4 °C. The plates were washed in phosphate buffer saline (1×) containing 0.05% tween 20 (washing buffer). Then the unbound proteins were blocked with 300 µl per well of 2.5% skim milk diluted in washing buffer, and then incubated at 37 °C for 1 h. After washing, 100 µl of each serum sample (diluted 1:100) in phosphate buffer saline (1×) was added to each well and the plates were incubated at ambient temperature for 1 h. The plates were then washed and incubated at ambient temperature for 1 h with anti-human-IgG horseradish peroxidase-conjugated antibody (Sigma-Aldrich, St. Louis, MO, USA) diluted in washing solution at 1:60,000. This was followed by washing of the plates and addition of 100 µl of substrate solution (3,3′,5,5-tetramethylbenzidine) (Invitrogen, Grand Island, USA) into each well. The reaction was stopped after 10 min of incubation by adding 50 µl per well of 2N sulfuric acid. The plates were read in a microplate reader at 450 nm to obtain the absorbance values of the protein.

### Dot blot immunoassay

PVDF membrane 0.2 µm (GE Healthcare, Chicago, Illinois, USA) was cut and soaked in methanol. The membranes were placed on a flat clean surface and clipped to the surface by needles. SCA (5 μl) was placed on identified spots on each membrane. After the antigen has been completely absorbed by the membranes, the membranes were incubated for 1 h at 37 °C in blocking buffer (50 mM Tris–HCl, 150 mM NaCl, 0.1% Tween-20). They were then washed 3 times each for 10 min in Tris buffer saline (1×) containing 0.1% Tween-20. The membranes were afterward incubated with 5 μl sera samples from *S. mansoni* infected and non-infected individuals for 30 min at room temperature. The membranes were washed and then incubated for 30 min with anti-human IgG peroxidase-conjugated antibody, diluted 1:10,000 in blocking buffer. This was followed by washing and revelation using ECL Plus Detection System (GE Healthcare, Chicago, Illinois, USA) and images were captured using chemiluminescence detection in ImageQuant LAS 4000 (GE Healthcare, Chicago, Illinois, USA).

### Western blot immunoassay for crude antigen and immunoreactive protein band

Two 12% SDS-PAGE were prepared. Five micrograms (5 μg) of SCA were loaded into each well for SDS-PAGE staining and Western blot. The proteins were separated using the Mini-Protean III (Bio-Rad), first at 60 V, and then 120 V for the coomassie staining gel. Proteins in the second gel were transferred electrophoretically to PVDF membrane 0.2 µm (GE Healthcare, Chicago, Illinois, USA) using a Mini-Trans-Blot (BioRad, Hercules, California 94547, USA) at 100 V (2–3 mA cm^2^) for 2 h 30 min at 4 °C with transfer buffer (25 mM Tris-Base, 205 mM glycine, 20% ethanol). The membrane was blocked overnight in 1 M Tris–HCl pH 7.5, 2.5 M NaCl containing 0.05% Tween-20 and 5% skim milk at 4 °C and then washed three times at 10 min/wash, in 10 mM Tris HCl. The membrane was gently cut into two for Western blot analysis for *S. mansoni* positive and negative sera samples. Each cut membrane was incubated in an immunoblotting buffer (1 M Tris–HCl pH 7.5, 2.5 M NaCl, 0.05% Tween-20, 5% skim milk) at room temperature under shaking for 20 min. Each membrane was individually incubated for 3 h in pooled *S. mansoni* positive and negative human sera samples diluted 1:500 in an immunoblotting buffer. The buffer was discarded after incubation, then the membranes were washed. The membranes were thereafter incubated under shaking at room temperature for 1 h 30 min with anti-human IgG peroxidase-conjugated antibody, diluted 1:2000 in the immunoblotting buffer. After three washes at 10 min/wash, the immunoreactive proteins were developed using ECL Plus Western Blotting Detection System (GE Healthcare, Chicago, Illinois, USA) and images were captured using chemiluminescence detection in ImageQuant LAS 4000 (GE Healthcare, Chicago, Illinois, USA)^16^. The Western blot analyses were performed in triplicate.

The Western blot protein analysis and its corresponding coomassie stained SDS-PAGE were overlapped to the dot blot analysis to identify the immunoreactive protein band. The marker protein band was excised from the SDS-PAGE gel. A total of 4 similar diagnostic marker protein bands from 4 lanes of the SDS-PAGE were pooled together in an Eppendorf tube and suspended in dimethylformamide solution for protein elution. The gels containing the protein were incubated for 16–18 h in a shaker incubator at 40 °C and 180 rpm. The gels were then removed and the tube content was centrifuged at 4000×*g* for 10 min. The supernatant was collected and the protein content was determined by the BCA method. Dot blot analysis was performed on 10 *S. mansoni* positive and 10 negative samples as previously described.

### Ethical considerations

Ethical approval for use of the samples was granted by the Ethics Committee of the René Rachou Institute, Fiocruz, Belo Horizonte, Brazil (CEPSH/CPqRR 03/2008). All humans experiments were carried out following the Code of Ethics of the World Medical Association (Declaration of Helsinki). Written informed consent was obtained from all participants.

### Statistical analysis

Data generated were analyzed by GraphPad Prism, version 5.0. The student’s t-test was used to determine a significant difference in IgG levels between *S. mansoni* infected and non-infected individuals. Results were presented as mean ± SD. Receiver operating characteristic curves (ROC curves), the area under curve (AUC), sensitivity, specificity, and the diagnostic cut-off point were determined by GraphPad Prism software version 5.0. The AUC is a measure of the accuracy of a quantitative diagnostic test. In general, AUC was interpreted as follows; 0.5 suggests no discrimination, 0.7–0.8 is acceptable, 0.8–0.9 is excellent, and ≥ 0.9 is considered outstanding^25^. Accuracy was determined by the formula; (number of true positives + number of true negatives)/(number of true positives + true negatives + number of false positives + number of false negatives). The sensitivity and specificity of SCA-ELISA were determined using GraphPad Prism statistical software. The cut-off point to discriminate patients with or without schistosomiasis was determined at maximum sensitivity and specificity also with the statistical software. The sensitivity of dot blots SCA assay was however computed using the formula; (number of true positives)/(number of true positives + false negatives). The specificity was computed using the formula; (number of negatives)/(number of true negatives + false positives). The degree of concordance between the SCA-ELISA and other diagnostic methods (KK and SG) was determined using kappa index (κ) and was then categorized as follows: < 0 poor, 0.00–0.20 slight, 0.21–0.40 fair, 0.41–0.60 moderate, 0.61–0.80 substantial and 0.81–1.00 almost perfect^26^. The statistically significant level was set at *p* < 0.05.

## Supplementary Information

Supplementary Information.

## Data Availability

The datasets during and/or analyzed during the current study available from the corresponding author on reasonable request.
