# Supplementary figures and images for: Evaluation of schistosomula crude antigen (SCA) as a diagnostic tool for Schistosoma mansoni in low endemic human population
Source: Sci Rep. 2021 May 18;11:10530. doi: 10.1038/s41598-021-89929-3 (PMC8131376; doi:10.1038/s41598-021-89929-3)

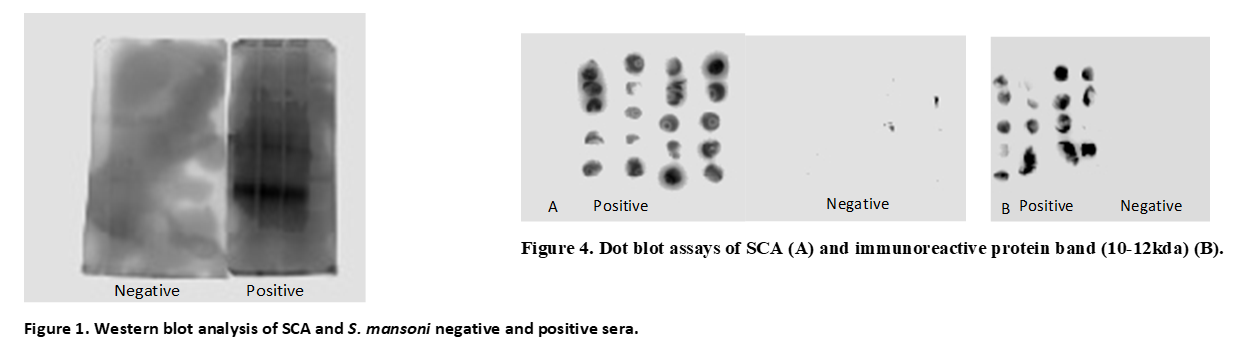

Supplement: Supplementary file 1 — Supplementary Information. [file 41598_2021_89929_MOESM1_ESM.tif]
